# The cost of Mycobacterium avium complex lung disease in Canada, France, Germany, and the United Kingdom: a nationally representative observational study

**DOI:** 10.1186/s12913-018-3489-8

**Published:** 2018-09-10

**Authors:** S. M. Goring, J. B. Wilson, N. R. Risebrough, J. Gallagher, S. Carroll, K. J. Heap, M. Obradovic, M. R. Loebinger, R. Diel

**Affiliations:** 1 ICONplc, Vancouver, Canada; 2 ICONplc, Toronto, Canada; 3 Clarity Pharma Research LLC, Spartanburg, USA; 4 Insmed Germany GmbH, Frankfurt, Germany; 5 Host Defence Unit, Royal Brompton Hospital and National Heart and Lung Institute, Imperial College London, London, UK; 6 Institute for Epidemiology, University Medical Hospital Schleswig-Holstein (Member of the German Center for Lung Research [ARCN]), Kiel, Germany

**Keywords:** Nontuberculous mycobacterium, Non-tuberculous mycobacteriosis, Observational study, Cost of illness, Direct medical costs

## Abstract

**Background:**

Management of nontuberculous mycobacterial lung disease (NTMLD) consists of a long-term multi-drug antibiotic regimen, yet many patients do not achieve culture conversion. We estimated the NTMLD-related direct medical costs in Canada, France, Germany, and the United Kingdom (UK) among refractory patients who were infected with *Mycobacterium avium* complex (MAC), without concomitant cystic fibrosis, tuberculosis, or HIV.

**Methods:**

We conducted a retrospective observational physician survey of nationally representative samples. The survey captured anonymized information about patients’ treatment histories for NTMLD-related health care resource utilization over a 24-month period. We summarized NTMLD-related resource use and estimated the total economic burden, from each country’s health care payer perspective.

**Results:**

In total, 59 physicians provided data on 157 patients. The average person time observed during the 24-month period was 1.7 years (SD: 0.4); 17% of patients died by the end of the study period. The major components of NTMLD-related direct medical costs among refractory patients were hospitalizations (varying from 29% of total annual costs in the UK to 69% in France), outpatient visits (8% in Canada to 51% in the UK), and outpatient testing such as post-diagnostic sputum testing, bronchial wash/lavage, spirometry, biopsies, imaging, and electrocardiograms (5% in France to 35% in Canada). In this patient cohort, the average direct medical costs per person-year, in local currencies, were approximately $16,200 (Canada), €11,600 (Germany), €17,900 (France) and £9,700 (UK).

**Conclusions:**

Based on this study’s findings, we conclude that managing patients with refractory NTMLD caused by MAC is associated with a substantial economic burden.

**Electronic supplementary material:**

The online version of this article (10.1186/s12913-018-3489-8) contains supplementary material, which is available to authorized users.

## Background

Nontuberculous mycobacterial lung disease (NTMLD) is a rare but emerging global health concern caused by mycobacteria that are ubiquitous in soil and water. Individuals with weakened immune systems or pre-existing structural lung damage tend to be at greatest risk of developing NTMLD, but it can also occur in patients without clear predispositions [1].

There is considerable geographic variability in the prevalence of NTMLD. In Europe, estimates range from 2 to 6 per 100,000 population, whereas in North America, estimates tend to be higher [2–7]. The distribution of the causative mycobacterial species also varies geographically. Species belonging to a group of slow-growing NTM, *Mycobacterium avium* complex (MAC), are the most common organisms associated with pulmonary disease [1], but they tend to be more prevalent among individuals with NTMLD in North America than in Europe [1, 8–11].

NTMLD typically presents as either nodular bronchiectasis, or cavitary disease. Depending on the presentation, symptoms can include persistent cough, fatigue, dyspnea, hemoptysis, fever, and unintended weight loss; chronic lung infection can lead to progressive damage to the lungs. Often, these symptoms interact with other pre-existing symptoms from concomitant diseases, such as bronchiectasis and chronic obstructive pulmonary disease (COPD), posing a significant burden on patients’ quality of life [12]. Moreover, many patients with MAC lung disease can have poor survival prognosis, with five-year all-cause mortality exceeding 30% in some studies [9, 13–15].

Recommended treatment of NTMLD consists of a long-term multi-drug antibiotic regimen [16]; however, these treatments often fail to convert cultures to negative or eradicate the infection. Sustained eradication of the microorganism is only achieved in 40% to 60% of patients, and 10% to 30% of patients discontinue their treatment due to adverse events [2]. Patients who are refractory to first-line therapy have limited therapy options, making them a particularly challenging population to treat successfully [2].

Little is known about the economic burden of NTMLD, although from the few existing estimates, the direct medical costs associated with managing NTMLD are thought to be substantial [7, 17–20]. To our knowledge, no data are available that specifically characterize the burden among those who are refractory to treatment.

To help inform resource allocation and priority setting by healthcare decision-makers, we sought to estimate the cost of MAC lung disease in Canada, France, Germany and the United Kingdom (UK), focusing on refractory patients without concomitant cystic fibrosis, tuberculosis, or HIV.

## Methods

We conducted a retrospective observational survey of nationally representative samples of physicians in Canada, France, Germany, and the UK to collect health care resource utilization data and associated costs among patients who were refractory to treatment for MAC lung disease, over a 24-month period. The choice of countries reflected the three most populous countries in Europe as well as Canada, where economic data play an important role in healthcare decision making process.

For a study to accurately represent its target population, each potentially qualified patient’s probability of study selection must be known and accurately represented in study findings. We met these requirements by: 1) identifying the population size of the active, patient-care physicians in NTMLD-treating specialties in each country, using as a guide the results of a comprehensive project by the EphMRA Foundation [21] to obtain accurate physician statistics for Canada, France and Germany; 2) using a master list containing contact information of physicians in each specialty, developed and continually updated by an international physician research supplier (Medefield) and supplemented by physician-contact information obtained from previous market research studies [5, 6] and the Nontuberculous Mycobacteria European Network Trials Group (NTM-NET); 3) contacting a random sample of physicians in NTMLD-treating specialties from each master list (between September 18 and December 24, 2015) and asking those who had managed a study-qualified patient within the past 24 months to complete an online survey, retrospectively using data from eligible patients’ charts; and 4) adjusting for over- or under-representation of the sampled population against the expected distribution of the population of NTMLD patients by applying an approach similar to propensity score weighting (see detail in Additional file 1). The factors influencing a patient’s weight were: 1) specialty of the treating physician, 2) patient volume of the treating physician and for the pooled analysis across countries, and 3) country-specific NTMLD prevalence. The expected distributions of these characteristics in the target population were informed by previous market research studies and literature-based estimates [5, 6].

The survey captured anonymized information about treatment history for NTMLD-related health care resource utilization. Physicians were permitted to extract information for up to 5 eligible patients (and were asked to select their most recently treated patients, if more than 5 were eligible), living or since deceased, who met all of the eligibility criteria: 1) infected with MAC: *M. avium, M. intracellulare*, and/or *M. chimaera*; 2) were considered refractory (defined as receiving at least 6 months of antimycobacterial treatment with a reasonable level of treatment compliance AND demonstrated at least one positive culture after at least 6 months of therapy); 3) received some or all treatment any time after September 2013 (24 months before the survey launch date); 4) no cystic fibrosis or tuberculosis in the past 24 months; 5) no HIV infection; 6) primarily under the responding physician’s care for management of NTMLD and not referred to another physician for management.

**Fig. 1:**
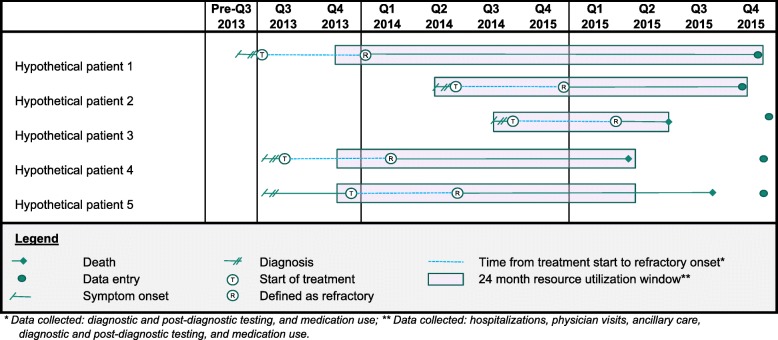
Study design.

Physicians provided detailed data relating to resource utilization (hospitalizations, physician visits, ancillary care, and other testing) during the 24 months prior to survey completion. Additionally, physicians provided data regarding diagnostic and post-diagnostic testing for NTMLD and medication use from the time of symptom onset to the time of survey completion (Fig. 1).

The questionnaire was programmed into an online format with safeguards to prevent missing data and disallow entries outside of reasonable pre-set ranges (determined in physician pre-testing and with the aid of literature review).

We summarized demographic and clinical characteristics of the refractory NTMLD patients infected with MAC. We quantified NTMLD-related resource use according to the proportion of subjects with any use, as well as the mean and standard deviation (SD) annual use per patient who used the resource. Person-time denominators were calculated based on the duration of time the patient was alive following the onset of NTMLD symptoms, up to a maximum of 24 months (because resource utilization was collected only for the 24 month period prior to survey completion). Dosing and duration of medication use during the two-year period were included in estimating total costs for oral antibiotics, IV antibiotics, and inhaled antibiotics.

We collected country-specific unit costs from publicly available sources, and literature review. Costs were taken from a health care payer perspective using 2015 € (France and Germany), $ (Canada) and £ (UK). Additional details on unit costing, along with all unit costs, are provided in Additional file 2.

In the main analysis, direct medical costs were calculated using two approaches. First, country-specific unit costs were applied to all components of resource utilization to estimate the annual direct medical cost per patient, for each country, expressed using local currencies. Second, country-specific total annual direct medical costs were converted to a common currency (2015 £), using the Organisation for Economic Cooperation and Development 2015 purchasing power exchange rates [22].

Given that some patients were eventually able to convert to negative and show clinical improvements, despite having been defined as refractory earlier in their course of treatment (specifically, after 6 months of treatment), we conducted an exploratory analysis to investigate direct medical costs among patients during two distinct phases: while having positive sputum cultures, and from the time of first negative sputum culture along with clinical improvement. In this exploratory analysis, data were pooled across countries, and overall resource utilization costs were re-calculated by applying UK unit costs to all patients’ resource utilization (UK unit costs; 2015 £). Not all patients contributed data to both phases: for example, some patients never had a negative sputum culture with clinical improvement.

A second exploratory analysis investigated drivers of annual direct medical costs using log-transformed cost data in simple and multiple linear regression models. As with the previous exploratory analysis, we re-calculated patients’ total costs by applying UK unit costs to all patients, allowing us to evaluate the influence of patients’ and treating physicians’ characteristics on total costs incurred, rather than simply revealing inter-country differences in unit costs. Inter-country management patterns remained a potential predictor of costs and thus country was included as a variable of interest. Other variables considered potentially related to cost were identified based on a brief literature review and clinical opinion.

All analysis was done using R version 3.4.1 [23]. An institutional review board reviewed the study and verified it as exempt due to the lack of identifiers, use of existing data, and no direct contact with patients (45CFR46.101(b)(4): Existing Data & Specimens - No Identifiers). Insmed, Incorporated funded the study.

## Results

In total, 59 physicians provided data (18 from Canada; 12 from France; 13 from Germany; 16 from the UK), on a total of 157 patient cases. Approximately 40% of physicians specialized in pulmonology/respiratory medicine, 20% specialized in infectious diseases, and most of the remaining 60% specialized in general medicine or internal medicine (Table 1).

**Table 1:** Physician characteristics

Physician characteristics	Number physicians = 59	
Country - *n* (%)		
Canada	18	(30.5)
France	12	(20.3)
Germany	13	(22.0)
United Kingdom	16	(27.1)
Primary specialty - *n* (%)		
General medicine	9	(15.3)
Infectious disease	12	(20.3)
Internal medicine	12	(20.3)
Pulmonology/respiratory medicine	24	(40.7)
Other^a^	2	(3.4)
Number of NTMLD patients managed in the last 24 months, per physician		
Mean (standard deviation)	20.3	(7.6)
Median (range)	20.0	(1–30)
Number of NTMLD patients included in study, per physician		
Mean (standard deviation)	2.6	(1.5)
Median (range)	3	(1–5)

^a^Nephrologist; neurologist

When pooling the sample across countries, weighted patient counts were: 36 from Canada; 36 from France; 47 from Germany; and 38 from the UK. The patients were mostly male (73%), with an average age at diagnosis of 58 years (SD: 14). Just over one third of patients had comorbid bronchiectasis (40.8%) (Table 2). We did not detect major differences in patient demographics across countries (data not shown).

**Table 2:** Patient characteristics

Patient characteristics	Number patients = 157	
Country - *n* (%)		
Canada	36	(22.9)
France	36	(22.9)
Germany	47	(29.9)
United Kingdom	38	(24.2)
Sex - *n* (%)		
Male	115	(73.2)
Female	41	(26.1)
Average age at diagnosis - mean (standard deviation)	58.4	(14.3)
History of smoking - *n* (%)		
Yes	115	(73.2)
No	36	(22.9)
Unknown	5	(3.2)
Vital status at end of study period - *n* (%)		
Alive	130	(82.8)
Deceased	27	(17.2)
Comorbid conditions - *n* (%)		
Bronchiectasis	64	(40.8)
Clinical characteristics of NTMLD at diagnosis - *n* (%)		
Nodular or cavitary opacities recorded on chest radiograph	67	(42.7)
Multifocal bronchiectasis with multiple small nodules on HRTC	56	(35.7)
HRCT scan shows cavities/nodular change	50	(31.8)
NTMLD species - *n* (%)^a^		
*M. avium*	101	(64.3)
*M. intracellulare*	59	(37.6)
*M. chimaera*	6	(3.8)
Other	3	(1.9)

^a^Values add to > 100% as patients may have been infected with more than one species. *Abbreviations*: *HRCT* high resolution computed tomography, *NTMLD* nontuberculous mycobacterial lung disease

Eighty-seven percent of subjects were followed for more than 1 year, and slightly more than half of patients were followed for over 2 years. (Table 3) The reasons for having less than 2 years of follow-up time were: NTMLD onset after the beginning of the two-year resource collection period (48% of patients), or death prior to the end of the study period (9% of patients for whom the death date was known; overall, 17% of patients were reported to have died by the end of the study period). The average person time observed during the two-year period was 1.7 years (SD: 0.4).

**Table 3:** Characteristics of follow-up time

	Number patients = 157	
Duration of follow-up, symptom onset to end of study period - *n* (%)		
Less than one year	20	(12.7)
Greater than or equal to one year; less than two years	56	(35.7)
Two years or more	80	(51.0)
Duration of observation during two year resource collection period - *n* (%)		
Duration of observation		
*Less than one year*	20	(12.7)
*Greater than or equal to one year; less than two years*	62	(39.5)
*Exactly two years*	75	(47.8)
Reasons for truncated observation period		
*Symptom onset after start of collection period*	75	(47.8)
*Died prior to end of collection period*^*a*^	14	(8.9)
Duration of follow-up, in years – mean (standard deviation)		
Time from symptom onset to initial diagnosis of NTMLD	0.4	(0.6)
Time from initial diagnosis to end of study period or death	2.1	(1.7)
Time from initial diagnosis to treatment start	0.3	(0.7)
Observation time during prior 24-month study period	1.7	(0.4)
Duration of follow-up, in years – median (range)		
Time from symptom onset to initial diagnosis of NTMLD	0.3	(0–5)
Time from initial diagnosis to end of study period or death	1.6	(0–13)
Time from initial diagnosis to treatment start	0.1	(0–6)
Observation time during prior 24-month study period	1.9	(0–2)

^*a*^and death date was known. *Abbreviations*: *NTMLD* nontuberculous mycobacterial lung disease

During the two-year period, 31.8% of patients had one or more pulmonary exacerbations; country-specific proportions ranged from 14.9% (Germany) to 47.2% (Canada) (Table 4). Exacerbations were accompanied by a spike in resource utilization: to treat the pulmonary exacerbation, 70.0% required a visit to the emergency room, 66.0% were hospitalized (mean LOS = 7.9 days; SD = 8.6), and 38.0% required a physician visit. Country-specific estimates of exacerbation-related utilization among the full study sample are presented in Table 4.

**Table 4:** Summary of country-specific resource utilization in the 24 month observation period

		Patients with any use (prior 24 months)							
		Canada		France		Germany		United Kingdom	
		*n* = 36		*n* = 36		*n* = 47		*n* = 38	
	Unit type	% with any use	mean units used ^a^	% with any use	mean units used ^a^	% with any use	mean units used ^a^	% with any use	mean units used ^a^
Pulmonary exacerbations									
Exacerbations that required ER visits	Visit	41.7	1.0	11.1	0.8	8.5	1.4	31.6	1.3
Exacerbations that required hospitalizations	Days	27.8	8.7	13.9	25.7	12.8	9.3	31.6	6.9
Exacerbations that required outpatient treatment									
Visit in physician’s office	Visit	19.4	2.5	16.7	2.5	2.1	1.4	13.2	1.2
IV infusion clinic	Visit	8.3	2.2	0.0	–	0.0	–	0.0	–
Other	Visit	0.0	–	0.0	–	0.0	–	0.0	–
Any pulmonary exacerbation	N/A	47.2	–	36.1	–	14.9	–	34.2	–
Other health care visits (not related to pulmonary exacerbations)									
ER visits	Visit	36.1	1.0	16.7	1.2	14.9	0.9	10.5	1.3
Hospitalizations									
Hospitalization for respiratory infection or inflammation	Days	44.4	5.4	86.1	12.8	72.3	6.7	55.3	7.9
Hospitalization requiring surgery	Days	0.0	–	11.1	0.7	0.0	–	2.6	8.0
Outpatient visits									
Physician office visits	Visit	91.7	5.8	61.1	9.5	51.1	3.8	52.6	4.0
Infusion clinic visits	Visit	2.8	7.5	0.0	–	6.4	3.5	0.0	–
Homecare nurse	Visit	16.0	70.9	44.2	27.7	22.8	60.5	1.3	0.4
Physiotherapy	Visit	30.4	24.3	59.7	19.4	33.9	10.1	25.5	12.3
Pulmonary rehabilitation	Visit	30.1	21.1	6.2	7.7	51.0	13.1	58.7	23.8
Other	Visit	10.6	15.4	7.4	48.8	5.3	9.7	2.1	50.4
Tests									
Biopsy	Test	4.7	9.2	25.0	1.9	30.7	10.0	8.6	2.2
Sputum specimen	Test	74.6	1.4	72.0	2.0	68.1	1.4	31.6	1.0
Bronchial wash or lavage	Test	23.7	1.8	66.0	1.0	47.4	1.3	76.5	1.6
Blood test	Test	52.7	6.0	86.7	6.9	70.6	6.3	76.6	6.1
CT scan	Test	74.5	2.3	75.1	6.7	54.9	9.7	65.7	2.4
ECG	Test	28.7	2.4	62.5	7.2	43.0	5.2	34.5	2.1
MRI	Test	8.3	5.6	18.5	6.4	11.1	4.9	1.3	1.6
Angiography	Test	0.0	–	0.0	–	4.5	1.1	0.0	–
CT guided biopsy	Test	0.0	–	0.0	–	4.5	3.5	8.6	1.3
Pulmonary function test	Test	24.6	1.6	14.1	1.2	46.4	5.0	28.8	1.6
Ultrasound	Test	1.4	0.8	19.9	5.9	39.2	4.6	2.8	1.5
X-ray	Test	73.9	3.9	69.5	7.8	73.1	3.8	75.0	3.9
Other	Test	10.6	1.4	0.0	–	7.9	7.3	0.0	–

^a^Mean annual utilization among subjects with any utilization of that resource within the prior 24 months. *Abbreviations*: *CT* computed tomography, *ECG* electrocardiogram, *ER* emergency room, *IV* intravenous, *MRI* magnetic resonance imaging

Overall, the major components of NTMLD-related direct medical costs among refractory patients infected with MAC were hospitalizations (varying from 29% of total annual costs in the UK to 69% in France), outpatient visits (8% in Canada to 51% in the UK), and outpatient testing (5% in France to 35% in Canada). Individual components of each of these cost categories are provided in Table 4.

**Fig. 2:**
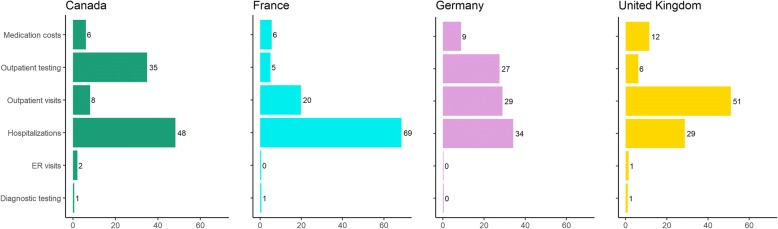
Component costs of NTMLD management: percentage of total direct NTMLD-related medical costs. Note: Types of visits and tests captured within the categories, ‘outpatient tests’ and ‘outpatient visits’ are listed in Table 2

Medication costs within this cohort of refractory patients did not contribute substantially to the overall burden of illness, ranging from 6 to 12% of total direct medical costs. (Fig. 2) The mean proportion of time exposed to medication during the two-year resource utilization collection period (following symptom onset and prior to death, where applicable) was only 64% (SD: 29%), indicating that many patients had discontinued medication prior to the end of the study period, or started after the period began.

There was some evidence of inter-country differences in the typical setting for medical care visits (Table 4). Hospitalizations were more common in France (where 86.1% of patients were hospitalized for respiratory infection or inflammation) than in Canada (where only 44.4% were hospitalized). Conversely, more than twice as many Canadian patients visited an ER during the two-year period compared with those in France (36.1% versus 16.7%), and almost all Canadian patients had at least one office-based physician visit (91.7%) compared with just over half of patients in the European countries (51.1% to 61.1%).

NTMLD testing patterns also differed across geographies (Table 4). Sputum specimen testing during follow-up tended to be more common in Canada (74.6%) than was reported in the UK (31.6%), yet the inverse trend was evident for bronchial wash or lavage (23.7% in Canada versus 76.5% in the UK). (Table 4).

**Fig. 3:**
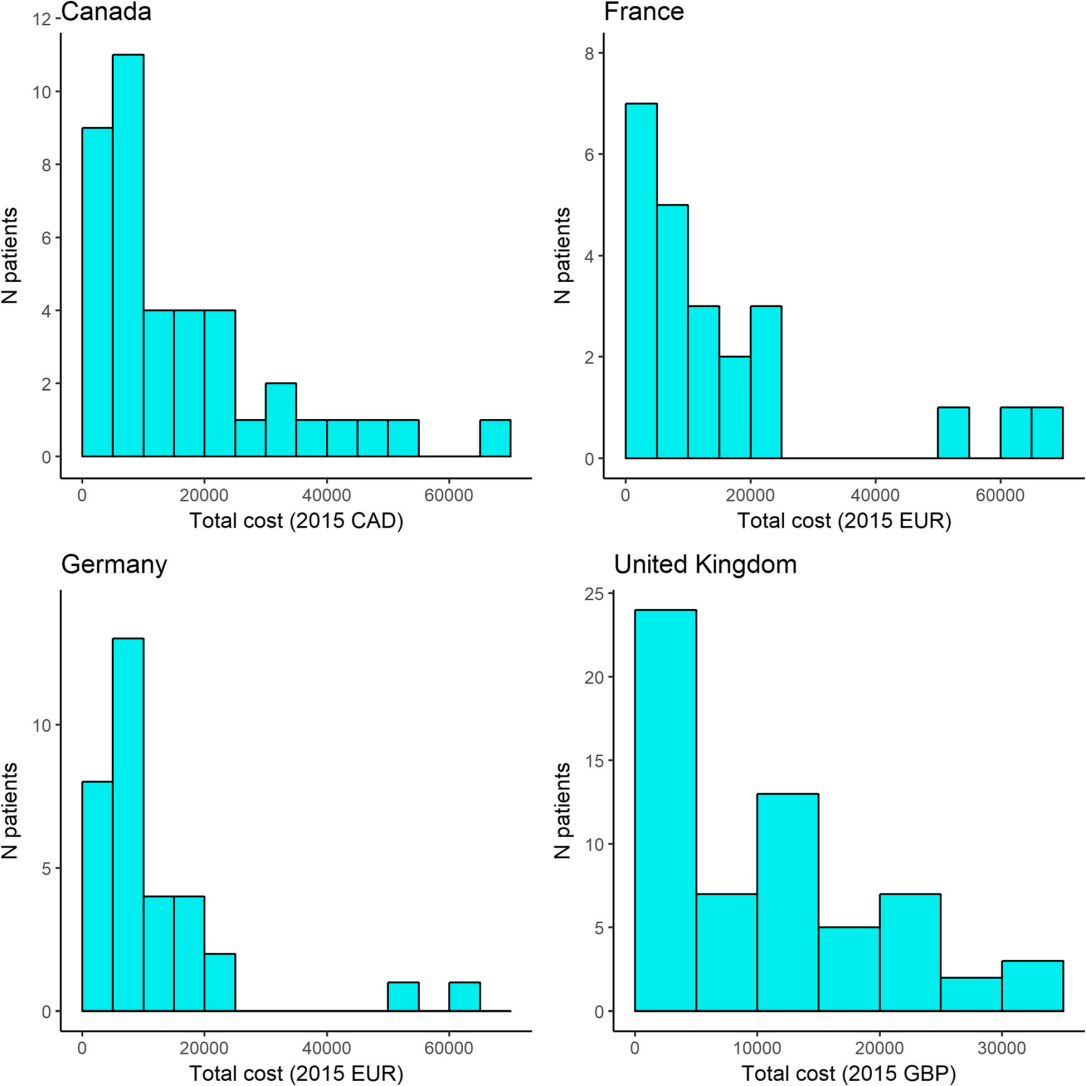
Histogram of cost distributions, by country (unweighted)

Overall, in this cohort of NTMLD patients infected with MAC who were refractory to treatment, the average direct medical costs per person-year were $16,209 (Canada), €11,626 (Germany), €17,881 (France) and £9,727 (UK) (Table 5). When converted to UK currency using purchasing power-based exchange rates, the average direct medical costs per person-year for Canada, France and Germany were £9,300, £15,264, and £10,434, respectively. Cost distributions were right-tailed (meaning that some patients had high costs): maximum costs were up to five times the mean costs (Fig. 3; Table 5).

**Table 5:** Annual NTMLD-related direct medical costs per patient in Canada, France, Germany, and the United Kingdom

Country	Annual direct medical cost per patient			
	Mean	Standard deviation	Median	Range
Local currency				
Canada (2015 $)	16,209	(15,168)	10,832	(808–66,503)
France (2015 €)	17,881	(17,449)	14,724	(2,145 - 68,907)
Germany (2015 €)	11,626	(13,330)	6,979	(884–61,558)
United Kingdom (2015 £)	9,727	(9,143)	7,454	(91–33,269)
Purchasing power parity conversion (£)				
Canada (2015 £)	9,300	(8,703)	6,215	(464–38,157)
France (2015 £)	15,264	(14,896)	12,569	(1,831 - 58,822)
Germany (2015 £)	10,434	(11,962)	6,263	(793–55,244)
United Kingdom (2015 £)	9,727	(9,143)	7,454	(91–33,269)

The first exploratory analysis, investigating direct medical costs while having positive sputum cultures, versus costs from the time of first negative sputum culture along with clinical improvement, revealed that average monthly costs were more than twice the cost – and median costs were triple – while testing positive than while testing negative (Additional file 3).

In the second exploratory analysis, investigating major cost drivers using regression models, it was apparent that the prevalence-based nature of the study cohort was influencing cost estimates. Subjects whose NTMLD diagnosis was incident during the observation window tended to have higher costs than those who had NTMLD onset prior to the beginning of the observation period. Using NTMLD onset date as an adjustment factor in the multiple regression model, physician specialty was the only characteristic that was significantly associated with NTMLD-related costs: patients treated by general practitioners were found to incur significantly greatest costs than those treated by infectious disease or pulmonary/respiratory medicine specialists. Additional detail on the cost regression is provided in Additional file 4.

## Discussion

This study captured detailed data on NTMLD-related resource utilization for a large group of patients with refractory NTMLD infected with MAC. The annual per-patient economic burden to health care payers was substantial, with country-specific estimates of $16,209 (Canada), €11,626 (Germany), €17,881 (France) and £9,727 (UK). These average costs were calculated among a prevalence cohort of patients during a two-year period, and during this period patients may have had either positive or negative sputum cultures. Multiplying these average annual costs by the prevalent population yields the expected excess cost that health care payer can expect to spend on treatment-refractory NTMLD patients infected with MAC.

While several studies investigating the economic burden of NTMLD have been conducted in the United States [7, 17, 19], to our knowledge, previous estimates are available from only two of the target countries considered in our study: Canada [18], and Germany [20]. In the previous Canadian study, the authors estimated a monthly economic burden of $499 (2008 $), which translates to an annual cost of $6,924 (2015 $). This is substantially lower than our own estimate (approximately $16,000); however, the former estimate excludes the costs of initial diagnostic investigations, emergency room visits, inpatient hospitalizations and surgeries; these component costs accounted for over 50% of the total costs in our study. Medication costs in the former study were higher than in our current study. This may be explained by two study design features. The first is the definition of person-time denominator. In the previous study, denominator person time was considered only *while on treatment*, whereas in our study, we considered time *while in the care of the physician (during the two-year period of resource use collection)*, regardless of whether or not treatment was used. Secondly, our study involved a cohort of refractory patients, and notably, more than half of subjects had discontinued treatment during the observation window.

In the study conducted in Germany [20], the annual costs of treatment attributable to NTMLD was just over €9,000, which is similar to our estimate, despite several differences in the study design and target population. Notably, the estimated health care costs in the German study were from an incidence-based cohort of subjects with NTMLD (based on an International classification of diseases [ICD]-10 code that captures all species of mycobacteria), and captured the excess health care costs relative to a matched comparison of subjects without NTMLD. In comparison, our study focused on a prevalence-based cohort of patients infected with MAC who became refractory to treatment, and estimated NTMLD-related costs by pre-defining the types of costs included. Despite these differences, both studies point to a substantial cost associated with the treatment and management of patients with NTMLD. Furthermore, both studies revealed relatively high mortality rates. The mortality rate in the German cohort was, on average, 6.9% annually over the 39-month study period [20], which is broadly similar to the mortality rate in the current study (17.2% over the 24-month study period, or 8.6% annually).

The restriction of the study sample to patients infected with MAC differs from most other studies examining the burden of NTMLD. There is evidence to suggest that costs of treating patients with *M. abscessus* and *M. xenopi* exceed the medical costs associated with treating patients with MAC, in particular due to more expensive medications and increased need for intravenous antibiotics for treating *M. abscessus* [17, 18]. *M. abscessus* is the most common NTM species among patients with concomitant cystic fibrosis, but is relatively less frequent in the full population of NTMLD [8, 24]. Thus, there may be some limitations in generalizing our findings among MAC patients to the broader population of refractory patients with NTMLD.

An additional differentiating feature of the patients included in our study, compared with other published burden of illness studies, was that they were considered refractory to primary treatment. These patients are more difficult to manage, have limited therapy options, and low treatment success rates [2, 25, 26]. Although half of the patients in our study were eventually able to convert to negative sputum culture and show clinical improvements, the other half remained sputum positive at the end of the observation period, and incurred greater costs.

A main strength of our research is the breadth and depth of data collection in this rare disease. The data for this study came directly from the charts of physicians who regularly manage NTMLD. The physicians were sampled from various settings, specialties, and geographic regions, and the provided data were adjusted to enable the sample to provide nationally representative results.

A core challenge of chart reviews is missing or incorrectly entered data. Certain gaps may exist due to incompleteness in charts, or periodic treatment by other physicians, and this is a limitation of the current study; however, the alignment of total costs in our study with other studies employing different methodologies and different data sources gives us confidence that the burden of NTMLD estimated in our study approximates the true economic burden to health care payers.

We enrolled a relatively small sample size of approximately 40 patients per country, and thus imbalances in the study population relative to the target population could influence the mean cost estimates – in particular given that the cost distribution reflects that there is a small number of patients incurring very high costs. Furthermore, the estimated resource utilization frequencies and cost comparisons across geographies may also have been impacted by the relatively small sample size. For example, the proportion of UK physicians using sputum specimens for post-diagnostic testing was unexpectedly low. Although it is possible that sputum testing is less commonly used among UK physicians than in other countries, the infrequent use may have been an anomaly among the sampled UK physicians, or could reflect incomplete records on sputum testing at these physicians’ sites. Similarly, inter-country differences in average annual direct medical costs should be interpreted with caution. Although the average annual cost per patient in France (converted to UK currency; approximately £15,000) was numerically higher than in Canada, Germany or the UK (approximately £9,000 to £10,000), in the multiple regression model, country was not a significant predictor of cost, which may in part reflect a lack of power for making such comparisons. We attempted to adjust for imbalances in patient recruitment by applying a weighting approach, and this was intended to improve the generalizability of our findings; however, without 100% capture of all patients, the risk of over- or under-representation remains.

Another limitation of the study is in estimating the costs of each unit of resource utilization to the health care payer. While costs were collected from country-specific governmental sources that publish health care costs, these costs represent averages across a wide variety of patients, not just those in our study population. Furthermore, in some cases, certain government payers do not provide coverage of services (e.g., physiotherapy in Canada) and therefore using a third-party payer perspective in our costing did not capture out-of-pocket costs or those incurred by private insurers. These differences in coverage of services, and in unit cost calculation for each country, is a limitation of the cost estimates and comparisons across countries; however, these are common challenges in most studies of economic burden, and we employed standard approaches to our costing, which represent the best approximation to the true costs.

The high economic burden of MAC-related NTMLD among treatment-refractory patients – largely driven by inpatient hospitalizations and frequent testing and follow-up – reflects an unmet need for effective treatments for successful and rapid eradication of NTMLD. In this study, we did not directly estimate the economic benefits of improved treatment success, adherence to therapy, or earlier initiation of therapy among newly diagnosed patients; however, this would be a natural extension to understand how to reduce the economic burden of NTMLD.

## Conclusions

Based on the findings of this study, we conclude that managing patients with refractory NTMLD caused by MAC is associated with substantial resource use, necessitating the need for improved patient management.

## Additional files

Additional file 1: Weighting strategy. Provides additional details and inputs for the weighting strategy employed in the study. (DOCX 24 kb)

Additional file 2: Unit costing. Includes country-specific unit costs used in the analysis. (DOCX 49 kb)

Additional file 3: Exploratory analysis 1. Supplemental methods and results of the first exploratory analysis, comparing direct medical costs while having positive sputum cultures, versus costs from the time of first negative sputum culture. (DOCX 18 kb)

Additional file 4: Exploratory analysis 2. Supplemental methods and results of the second exploratory analysis involving cost regression models. (DOCX 25 kb)

## Abbreviations

COPD: Chronic obstructive pulmonary disease
ICD: International classification of diseases
MAC: Mycobacterium avium complex
NTMLD: Nontuberculous mycobacterial lung disease
SD: Standard deviation
UK: United Kingdom
