# Supplementary material for: The cost of Mycobacterium avium complex lung disease in Canada, France, Germany, and the United Kingdom: a nationally representative observational study
Source: BMC Health Serv Res. 2018 Sep 10;18:700. doi: 10.1186/s12913-018-3489-8 (PMC6131733; doi:10.1186/s12913-018-3489-8)
Supplement: Supplementary file 1 — Weighting strategy. Provides additional details and inputs for the weighting strategy employed in the study. (DOCX 24 kb) [file 12913_2018_3489_MOESM1_ESM.docx]

**Additional file 1: Weighting Strategy**

The weighting strategy applied in this study has been previously published.[1-4]

For a survey to accurately reflect its target population, each participant’s probability of being selected from the universe of study-qualified patients must be known and accurately represented in the survey findings. This requires, for this study, knowing the size of the NTMLD patient universe, the percentage of all such patients in the universe managed by physicians in each treating specialty, and the number of NTMLD patients managed by each study-participating physician from whom the survey patient is selected.

A nationally representative survey of patients with a target condition requires that each randomly selected target patient’s probability of being selected from the target-patient universe be accurately reflected in study findings. For each country, we calculated each patient participant’s study-selection probability by determining (1) the probability that the patient’s treating physician would be study-selected (from the universe of patient-care physicians in the physician’s specialty) and (2) the individual patient’s proportion of the total number of target patients treated in the past 12 months by all physicians in the corresponding specialty. When a study patient’s probability of study selection was higher or lower than the patient’s study-selection probability, we applied a weighting factor that corrected (up-weighted or down-weighted) for the over- or under-representation of the corresponding observations.

The number of total weighted patients within each country parallels the total number of study patients for the country. A variable titled “Sur_Wt” was created in the study database that contains a weight for each patient in each target country.

Another type of weight was also developed to adjust for NTMLD population differences between study countries. This variable is titled “Epi_Wt”; it incorporates annual NTMLD prevalence estimates and is applied only to pooled between-country summaries. It enables the analysis of composite study findings for multiple countries by adjusting for NTMLD population differences between the four countries. Each patient’s Epi_Wt maintains the appropriate within country proportions for each patient but also provides for each patient’s proportion of the combined total sample (Canada, France, Germany, and United Kingdom).

The NTMLD population estimates used in weighting are contained in Table 1.

Table 1. Weighting calculations for treatment refractory NTMLD population

| **Country** | **Total 2016 population^1^** | **Country  % of combined 2016 total 4-country population** | **Estimated annual prevalence of NTMLD by country^2,3^** | **Estimated number of annual NTMLD patients by country** | **Estimated % of 2016 total 4-country NTMLD population** |
| --- | --- | --- | --- | --- | --- |
| **France** | 66,567,560 | 26.9% | 6.0 | 3,994 | 23.0% |
| **Germany** | 80,722,792 | 32.7% | 6.5 | 5,247 | 30.1% |
| **United Kingdom** | 64,430,428 | 26.1% | 6.5 | 4,166 | 23.9% |
| **Canada** | 35,362,905 | 14.3% | 11.3 | 3,996 | 23.0% |
| **Combined Total** | **247,083,685** | **100.0%** | **7.5** | **17,403** | **100.0%** |

*(1) Total 2016 population for each country obtained from U.S. Census Bureau, International Data Base (IDB), Source of Country Populations (http://sasweb.ssd.census.gov/cgi-bin/broker) (2) Prevalence estimates based on the 2014 Clarity NTMLD study.*

*(3) NTMLD prevalence and treatment refractory NTMLD estimates for Canada were based on an analysis of a study by Marras et al., who reported the annual prevalence of NTMLD disease in Ontario for the years 1998 to 2010. The authors hypothesized that there was an increase in prevalence over time, and tested the difference in 5-year prevalence between (1998 to 2002) and (2005 to 2010); the results of the study were consistent with their hypothesis. The authors also noted an attenuation in the rate of increase, from mid-study period, and the trend appears to be linear from that point. -For that reason, to estimate NTMLD prevalence at beginning of 2016, we assumed a linear trend and used data from the latter part of the study period (2005 to 2010) to estimate the average annual increase. ICON projected forward from the mid-point of that study period to 2016, which resulted in an annual prevalence estimate for NTMLD disease of 11.3 per 100,000 population.*

**References**

1. Gallagher, J., *A Cost-Effective Enhanced Retrospective Observational Study Methodology to Capture Economic Burden Evidence in a Rare Disease Using Nontuberculous Mycobacterial Pulmonary Disease (NTMPD) as a Model* in *ISPOR*. 2015: Milan, Italy.

2. Balkrishnan, R., et al., *Predictors of treatment choices and associated outcomes in actinic keratoses: results from a national physician survey study.* J Dermatolog Treat, 2006. **17**(3): p. 162-6.

3. Cookson, M.S., et al., *National practice patterns for immediate postoperative instillation of chemotherapy in nonmuscle invasive bladder cancer.* J Urol, 2012. **187**(5): p. 1571-6.

4. Palou-Redorta, J., et al., *The use of immediate postoperative instillations of intravesical chemotherapy after TURBT of NMIBC among European countries.* World J Urol, 2014. **32**(2): p. 525-30.
