# Supplementary material for: The cost of Mycobacterium avium complex lung disease in Canada, France, Germany, and the United Kingdom: a nationally representative observational study
Source: BMC Health Serv Res. 2018 Sep 10;18:700. doi: 10.1186/s12913-018-3489-8 (PMC6131733; doi:10.1186/s12913-018-3489-8)
Supplement: Supplementary file 2 — Unit costing. Includes country-specific unit costs used in the analysis. (DOCX 49 kb) [file 12913_2018_3489_MOESM2_ESM.docx]

**Additional file 2: Unit Costing**

**Hospital and Outpatient Visit Costs:**

Table 1. Unit costs for hospitalizations and health care visits

| **Visit type** | **Canada**  **(2015 CAN)** | **UK**  **(2015 £)** | **France**  **(2015 €)** | **Germany**  **(2015 €)** |
| --- | --- | --- | --- | --- |
| Homecare nurse visits | 42.36 | 37.00 | 43.34 | 41.23 |
| Physiotherapist visits | 0.00 | 78.00 | 91.37 | 86.91 |
| Pulmonary rehabilitation visits | 72.67 | 314.42 | 368.32 | 350.35 |
| Physician office visits | 38.05 | 149.14 | 23.00 | 60.37 |
| Intravenous infusion clinic visits | 281.73 | 161.65 | 189.36 | 180.12 |
| Other visits | 17.50^a^ | - | 454.68^b^ | - |
| Emergency room visit | 450.42 | 264.30 | 309.61 | 128.74 |
| Per Hospital Admission (by DRG) | - | - | 4,662.83^c^ | 3,237.69^d^ |
| Per Hospital Admission (by DRG), per excess day | - | - | Not applicable | 317.25^e^ |
| Per Hospitalization Day (by DRG) | 1,640.00 | 377.43 | 627.06^f^ | - |
| Per Surgical Admission (by DRG and Procedure) | - | - | 11,224.84 | See text |
| Per Surgical Admission (by DRG and Procedure), per excess day | - | - | Not applicable | 334.23^g^ |
| Per Surgical Admission Day (by DRG) | 2,765.00 | 1398.34 | - | - |

^a^Pharmacist visit; ^b^multidisciplinary hospital team single day hospital stay; ^c^the cost for hospitalisation in France was taken as a weighted average across all types of hospitalizations encoded for ICD-10 code A31.0 (pulmonary mycobacterial infection) based on the PMSI (French hospital stay database) 2014 analysis, inflated to 2015; ^d^the cost for up to 14 days LOS in Germany was taken as the cost for hospitalizations with DRG code E76C (Other diseases of the respiratory organs without extremely severe cc and with or without signs and symptoms of breathing with complex diagnosis)j; ^e^ Costs for every day over 14 days of hospitalization; ^f^ Single day hospitalizations in France only; ^g^Costs per excess day over 22 days stay.

**Hospital Costs:**

NTMLD-related hospital costs were determined for ICD10 A310 were incorporated per day stay in Canada and the UK and per hospitalization in France (weighted average of DRGs) and selected DRG per admission in Germany.

The cost of hospitalizations was included for exacerbations and lobectomies. No hospitalizations coded for A31.0 (NTM) were available from the Canadian hospital costing dataset at the Canadian Institute for Health Information. Thus Canadian hospital cost was based on J22 unspecified acute lower respiratory infection in 2010, inflated to 2015 and included a specialist/respirologist subsequent visit in hospital per day for a total cost of 1640.00 per day (OCCI 2010,[1] Statistics Canada 2015.[2]) Hospitalization for surgery (1GR87QB) was based on the cost of hospitalization to excise part lung lobe, open approach, including hospital and surgical procedure costs with a specialist/respirologist subsequent visit in hospital per day for a total cost of 2765.00 per day. Based on the OCCI data set the J22 hospitalization had an average length of stay of 5.6 days and the surgical procedure hospitalization had a mean stay of 7.6 days. Ambulatory care costs were also obtained from the 2006/2007 Alberta Case Cost Report. The cost of an ER visit was assigned the cost of managing severe respiratory disease (ACCS 864, $265), Alberta Case Costing Report, 2006/2007 and inflated to 2015. [3] The hospitalization and ambulatory care costs were then inflated to 2015 currency using the health care component.

In the UK, the weighted cost per day for a hospitalization (£377.42) was based on the average cost per admission and a weighted average of LOS from elective, non-elective and day case admissions for ‘Other Respiratory Disorders’. The average cost per day for surgery (£1398.30) was based on a weighted average LOS and cost per day for Complex Thoracic Procedures. [4] The cost per emergency contact was based on the weighted average cost of all Emergency medicine HRG 2013-2014, inflated to 2015 and included one emergency physician consultation fee for a total cost of £264.30. [4]

German hospital costs were based on admissions with ICD-10 code A.310 and DRG E76C (Other diseases of the respiratory organs without extremely severe CC or signs and symptoms of breathing with complex diagnosis) for NTMLD related hospitalizations and for surgery, DRG E05C (Other large operations on the thorax without extremely severe CC, except for malignant neoplasm) with procedure code 5-324.61. Selection of the relevant DRG for the NTMLD population was confirmed with a local treating physician based on historical analysis of coding of cases. Costs were calculated based on the cost per admission (up to 14 days, DRG E76C of €3,237.69 and an additional cost of €317.25 per day length of day in excess of 14 days in the Fallpauschalenkatalog, 2015 published by the InEK. Surgical cost by DRG applicable by maximum day stay was the following: €5,245.19 (1 day); €6,488.88 (2 days); €7,832.58 (3 days) €9,176.27 (4 to 22 days stay) and €334.23 per excess day above 22 days stay. [5, 6] Inpatient physician fees are included in hospital DRG costs. The cost per emergency contact taken from the 2014 German Expertise in Hospital Care (Gutachten zur ambulanten Notfallversorgung im Krankenhaus) and inflated to 2015 Euros; a total cost of £128.74. [4]

Data on public hospital activity and expenditures are available through the French DRG based information system called the Programme de Médicalisation des Systèmes d’Information (PMSI) Médecine, Chirurgie (surgery), Obstétrique (MCO).[7] In 2014, there were 802 patients with a total of 1193 hospitalizations in public hospitals related to the diagnosis A310 (pulmonary infection with atypical mycobacteria) in adults. Inflated to 2015 costs, the weighted average cost of the DRGs for all A310 hospitalizations was €4662.83 per admission. The weighted average of A310 hospitalization DRGs for surgical interventions (Major thoracic intervention level 2 or 3) was €11,224.84. Agence Technique de l’Information sur l’Hospitalisation 2015,[7] INSEE 2015. [8] Costs include fixed and variable costs. Fixed costs include physician and other salaries, devaluation and maintenance of medical equipment, restaurant and food, laundry and other logistics. Variable costs include medical consumable items, surgery, radiology, laboratory, anesthesiology, and other activities. In France, physicians frequently admit NTMLD patients for the day for follow-up and consultation, thus a large number of single day admissions were evident in the dataset. To reflect these practice patterns, “consultant visit hospital day admission” cost for a single day admission was set to (€627.06 per single day stay) based on the minimum cost in the range of the weighted of the A310 hospitalizations. The cost for an Emergency room (ER) visit was unknown, therefore the ER visit cost based in the UK (£264.30) was converted to France (€309.61) using the purchasing power parity.

**Outpatient visits:**

Outpatient physician fees were based on the Schedule of Benefits and inflated to 2015 Canadian dollars where applicable.[9] Physician office visits were based on an infectious disease or internal medicine subsequent visit (A468 or A138, $38.05) and the per day cost for homecare nursing care at home was $42.36. [10] A high percentage of physiotherapy visit costs are reimbursed by private payers or the patient; therefore, the cost for these visits from the perspective of the Ministry of Health was assumed to be $0.00. Pulmonary rehabilitation visits were based on a published report for costs of pulmonary rehabilitation for COPD. [11] The total cost for rehabilitation services was $1211 and the cost per visit was derived by assuming that patients would have 3 sessions per week for 6 weeks for a total of 18 visits; therefore, the cost per visit, (inflated to 2015) was $72.76.

In the UK, outpatient physician fees were based on the National Schedule of Reference Costs [4]. These fees were £37.00 for a face to face nurse visit at home (homecare nurse); £78.00 for an adult one on one physiotherapy visit; £314.42 for rehabilitation for respiratory disorders (pulmonary rehabilitation), £149.14 for a follow-up visit in general medicine (physician office visit), and £161.65 per visit to an outpatient parenteral antimicrobial therapy (OPAT) clinic, physiotherapist, based on a previously published estimate (46) and inflated to 2015 £ (47).

In Germany, outpatient physician fees were based on the Uniform assessment scale of the Kassenärztliche Bundesvereinigung (KVB) (Einheitlicher Bewertungsmaßstab).[12]. The outpatient physician fees were applied as a maximum cost per quarter for a patient with any number of visits in a three month timeframe (€60.37) which is in line with outpatient visit reimbursement regulations.

In France, outpatient physician fees were based on the tariffs provided by the Sécurité Sociale Assurance Maladie.[13] The cost for a general practitioner and specialist visit was €23.00 and €25.00, respectively.

**Drug Costs:**

Table 2. Unit costs for medications, cost per mg

|  | **Canada**  **(2015 CAN)** | **UK**  **(2015 £)** | **France**  **(2015 €)** | **Germany**  **(2015 €)** |
| --- | --- | --- | --- | --- |
| **Oral medications (per mg)** |  |  |  |  |
| Dispensing fee | 8.830 | - | 0.820 | - |
| Azithromycin | 0.005 | 0.002 | 0.006 | 0.009 |
| Ciprofloxacin | 0.001 | 0.000 | 0.002 | 0.002 |
| Clarithromycin | 0.002 | 0.000 | 0.002 | 0.003 |
| Clofazimine | 0.007 | 0.009 | 0.005 | 0.006 |
| Cotrimoxazole | 0.001 | 0.000 | 0.005 | 0.001 |
| Doxycycline | 0.000 | 0.001 | 0.002 | 0.003 |
| Ethambutol | 0.002 | 0.002 | 0.000 | 0.001 |
| Isoniazid | 0.007 | 0.007 | 0.003 | 0.001 |
| Levofloxacin | 0.005 | 0.003 | 0.005 | 0.003 |
| Linezolid | 0.000 | 0.074 | 0.005 | 0.006 |
| Minocycline | 0.007 | 0.004 | 0.004 | 0.004 |
| Moxifloxacin | 0.015 | 0.006 | 0.005 | 0.011 |
| Rifabutin | 0.029 | 0.020 | 0.023 | 0.031 |
| Rifampin | 0.003 | 0.001 | 0.002 | 0.006 |
| Other | 0.000 | 0.009 | 0.005 | 0.006 |
| **IV antibiotics:** |  |  |  |  |
| Ciprofloxacin IV | 0.051 | 0.049 | 0.070 | 0.072 |
| Levofloxacin IV | 0.211 | 0.048 | 0.041 | 0.096 |
| Tobramycin IV | 0.058 | 0.047 | 0.036 | 0.164 |
| Colistinmethate IV | 0.203 | 0.104 | - | 0.069 |
| Cefoxitin IV | 0.011 | 0.104 | 0.041 | 0.010 |
| Imipenem IV | 0.049 | 0.024 | - | 0.041 |
| Amikacin IV | 0.070 | 0.019 | 0.041 | 0.070 |
| Meropenem IV | 0.030 | 0.016 | 0.031 | 0.040 |
| Linezolid IV | 0.167 | 0.074 | - | 0.062 |
| Tigecycline IV | 1.428 | 0.646 | - | 0.069 |
| Other IV | 0.044 | 0.015 | - | 0.069 |
| **Inhaled antibiotics (per mg):** |  |  |  |  |
| Amikacin | 0.070 | 0.019* | 0.232 | 0.070*- |
| Aztreonam | 0.091 | 0.346 | 0.333 | 0.17 |
| Tobramycin | 0.091 | 0.071 | 0.131 | 0.17 |
| Colistin | 0.091 | - | 0.232 | - |
| Meropenem | 0.091 | 0.018 | 0.232 | - |
| Streptomycin | 0.091 | 0.015 | 0.232 | - |

*Abbreviations: IV = intravenous*

*Cost of IV amikacin used; Note: *For all drug costs, the lowest costs were assumed.*

In Canada, the costs of oral antibiotics were taken from the Ontario Drug Benefit (ODB).[14] The lowest available unit costs were assumed. The total daily dose for each antibiotic was based on the dose indicated by physicians. A separate dispensing fee is charged with all medications filled at an outpatient pharmacy which is $8.83 per prescription.[15] The cost of IV antibiotics was based on incorporating the cost of the medication. The unit cost of IV antibiotics was obtained from the Régie de l’assurance maladie du Québec (RAMQ) List of Medications.[16] Due to capturing the costs associated with IV infusion clinic visits elsewhere in the survey, administration costs were excluded, thus avoiding double counting. The inhaled antibiotic costs were not available from hospital formulary or public sources; therefore, a pragmatic approach was developed where the generic cost of Tobramycin ($0.091 per mg) was applied to all inhaled antibiotics. [14]

In the UK, oral and IV and inhaled antibiotics unit costs were taken from the British National Formulary.[17] Due to capturing the costs associated with IV infusion clinic visits elsewhere in the survey, administration costs were excluded, thus avoiding double counting.

In Germany, the costs of oral antibiotics were taken from the pharmacy level price (Apothekenverkaufspreis) list of fixed price drugs (either the Rote List if available or the Lauer Taxe) published by the National Association of Statutory Health Insurance Funds (Gesetzliche Krankenversicherung [GKV] Spitzenverband). [18, 19] The cost of IV antibiotics was determined by incorporating the cost of the medication. Due to capturing the costs associated with IV infusion clinic visits elsewhere in the survey, administration costs were excluded, thus avoiding double counting. An average price per ml or mg, respectively, was calculated, which was then used to calculate the cost per infusion. Drug costs were obtained from Lauer-Taxe, [19] and administration costs were based on the Einheitlicher Bewertungsmaßstab and infusion times were taken from the product monographs. The cost of inhaled antibiotics was obtained from Lauer-Taxe.[19] The average cost of inhaled tobramycin per mg was applied to aztreonam – the only other inhaled antibiotic utilized in Germany in the dataset.

In France, the costs of oral and IV antibiotics were taken from a public administrative database of medicinal products which has been put in place by the Agence Nationale de Sécurité du Médicament et des Produits de Santé (ANSM) in collaboration with the Haute Autorité de Santé (HAS) and the Union Nationale des Caisses d’Assurance Maladie (UNCAM) from the Ministry of Social Affairs and Health.[20] The cost of antibiotics did not take into account the dispensing fee of 0.82€ per prescription.[21] A dispensing fee of 0.82€ per package was included for IV antibiotics. [21] Due to capturing the costs associated with IV infusion clinic visits elsewhere in the survey, administration costs were excluded, thus avoiding double counting. In Canada and France dispensing fees are extra charges with the prescription, however in the UK and Germany no dispensing fees are charged and are included in the drug costs.

**Laboratory and Procedure Costs:**

Table 3. Unit costs for tests

|  | **Canada**  **(2015 CAN)** | **UK**  **(2015 £)** | **France**  **(2015 €)** | **Germany**  **(2015 €)** |
| --- | --- | --- | --- | --- |
| **Biopsy** |  |  |  |  |
| Lung | 725.74 | 151.00 | 249.57 | 610.27 |
| Blood test | 34.64 | 24.00 | 29.16 | 13.60 |
| Monitoring for amikacin | 34.64 | 24.00 | 29.16 | 13.60 |
| **CT** |  |  |  |  |
| Neck/thorax | 1396.66 | 103.00 | 25.27 | 146.78 |
| Neck/thorax/abdomen | 1989.03 | 100.00 | 25.27 | 230.91 |
| Thorax/abdomen/pelvis | 2086.01 | 100.00 | 25.27 | 220.02 |
| Other | 794.92 | 92.00 | 25.27 | 99.62 |
| ECG | 384.38 | 57.00 | 37.80 | 8.22 |
| **MRI** |  |  |  |  |
| Thorax | 643.75 | 145.00 | 69.00 | 124.60 |
| Thorax (contrast) | 643.75 | 166.00 | 69.00 | 148.02 |
| Angiography | 883.09 | 174.46 | 204.37 | 322.02 |
| CT-guided biopsy | 1455.71 | 881.21 | 209.00 | 126.24 |
| Pulmonary function test | 17.15 | 63.00 | 13.32 | 41.81 |
| **Ultrasound** |  |  |  |  |
| Abdomen | 581.46 | 52.00 | 37.80 | 28.86 |
| **X-ray** |  |  |  |  |
| Chest/thorax | 446.25 | 30.00 | 23.94 | 15.61 |
| Peripherally inserted central catheter | 883.09 | 174.46 | 23.94 | 322.02 |
| Clavicle | 262.19 | 30.00 | 23.94 | 14.48 |
| Other test (Sputum) | 11.37 | 6.52 | 24.90 | 7.20 |
| **Other tests** |  |  |  |  |
| Bronchial wash or lavage | 147.90 | 7.00 | 8.20 | 9.40 |
| Sputum specimens for acid-fast bacilli | 36.35 | 6.52 | 24.90 | 81.80 |
| Transbronchial or other lung biopsy | 262.00 | 642.00 | 249.57 | 126.85 |
| Chest high-resolution computed tomography scan | 75.85 | 0.00 | 25.27 | 67.79 |
| Chest radiograph | 21.65 | 30.00 | 23.94 | 15.61 |
| Tests to exclude tuberculosis | 89.85 | 202.00 | 59.00 | 8.15 |
| Other | NA | NA | 187.51 | 61.40 |

*Abbreviations: CT = computed tomography; ECG = electrocardiogram; MRI = magnetic resonance imaging*

In Canada, costs for laboratory tests were based on labour, materials, supervision (LMS) units as specified in the Schedule of Benefits for Laboratory Services[22] and were calculated based on the number of LMS units assigned to each test multiplied by the cost per LMS unit ($0.517). Costs for procedures included technician and physician fees from the Schedule of Benefits[9] and were taken from the 2013 publication of the Ontario Case Costing Initiative (OCCI) inflated to the 2015 Canadian dollar. Total costs were based on summing the cost of the procedure and all applicable technician and or physician fees. The cost of a CT guided biopsy was based on assuming the hospital cost of a lung biopsy (OCCI code: 2GT71HA) and CT scan of the thoracic cavity (OCCI code: 3GY20WA) and the physician fees for performing lung biopsies (SoB billing code: Z340). This assumption was made since there is no billing code for CT guided biopsy.

In the UK, laboratory procedure costs related to diagnosing NTM, confirming NTM conversion, and disease or complication monitoring were included in the analysis. Unit costs for these laboratory procedures were based on the National Reference Cost.[4]

In Germany, the cost of laboratory tests were based on the Uniform Assessment Scale of the KVB (Einheitlicher Bewertungsmaßstab).[12]. The cost related to ruling out TB following a sputum smear (€7.26) included a TB skin test (TST, €0.95); following sputum smear, the cost for mycobacterial culture growth (€34.90) and staining (€5.20) was considered. [12]. Costs of the procedures were taken from the Einheitlicher Bewertungsmaßstab.[12] The X-ray of the thorax is only priced for one side of the body; therefore, the cost was included twice to calculate a full thorax scan. Outpatient tests were included for biopsies, CT scans, MRI, ultrasounds, x-rays, electrocardiograms, PICC line insertion and removal, and spirometry tests.

In France, laboratory fees were based on the tariffs provided by the Sécurité Sociale Assurance Maladie. [23] Resource use and costs for tests and procedures performed in the hospital are available for the diagnosis A310 (pulmonary infection with atypical mycobacteria) from la Sécurité Sociale Assurance Maladie which classifies all acts with a Classification Commune des Actes Médicaux (CCAM) code. The acts are classified in order of importance from most frequent to least with associated costs. Bronchial wash or lavage cost is based on a converted relative UK cost using the pricing parity approach.

**References:**

1. *Ontario Case Analysis Tool*. Ministry of Health and Long-Term Care, 2013. Available from: <http://www.occp.com/mainPage.htm>.

2. Database, C.S.I.a.M. *Table 326-0020 Consumer price index monthly (2002=100)*. Statistics Canada, 2015. Available from: <http://www5.statcan.gc.ca/cansim/a26?lang=eng&retrLang=eng&id=3260020&paSer=&pattern=&stByVal=1&p1=1&p2=37&tabMode=dataTable&csid>=.

3. *Alberta Case Cost Report for 2006/2007 Hospital Activity*, in *Alberta Health and Wellness*. 2009, Alberta Health.

4. *NHS reference costs 2013 to 2014*. NHS, 2015. Available from: <https://www.gov.uk/government/publications/nhs-reference-costs-2013-to-2014>.

5. *Fallpauschalen-Katalog: G-DRG-Version 2014*. Institut für das Entgeltsystem im Krankenhaus (InEK), 2014. Available from: <http://www.aok-gesundheitspartner.de/imperia/md/gpp/bund/krankenhaus/drg_system/gdrg/fallpauschalenkatalog_2014_20131018.pdf>.

6. *Reimbursement info*. [Website] 2017 [cited 2017 June 7]. <https://app.reimbursement.info/drgs/E76C?year=20152017>

7. *MCO par diagnostic ou acte (hospital)*. Agence Technique de L'information sur l'Hospitalisation (ATIH), 2015. Available from: <http://www.scansante.fr/applications/statistiques-activite-MCO-par-diagnostique-et-actes>.

8. *Indice des prix a la consommation-Nomenclature COICOP 06.1-Medicaments, autres produits pharmaceutiques, appareils et materiels therapeutiques*. Institut National de la Statistique et des Etudes Economiques, 2015. Available from: <http://www.insee.fr/fr/bases-de-donnees/bsweb/serie.asp?idbank=000637734>.

9. *Schedule of benefit for physician services under the Health Insurance Act*. Ministry of Health and Long-Term Care, 2015. Available from: <http://www.health.gov.on.ca/english/providers/program/ohip/sob/physserv/physserv_mn.html>.

10. *Office of the Auditor General of Ontario Annual Report 2015*. 2015, Office of the Auditor General of Ontario: Toronto. Available from: <http://www.auditor.on.ca/en/content/annualreports/arbyyear/ar2015.html>

11. *Pulmonary Rehabilitation for Chronic Obstructive Pulmonary Disease*, in *Health Technology Assessment*. 2010, CADTH. Available from: <https://www.cadth.ca/sites/default/files/pdf/H0482_COPD_tr_e.pdf>

12. Bundesvereinigung, K. *Online-version des einheitlichen bewertungsmaßstabs (EBM)*. Kassenärztliche Bundesvereinigung, 2015. Available from: <http://www.kbv.de/html/online-ebm.php>.

13. *Les consultations en metropole (doctors tarifs)*. Securite Sociale Assurance Maladie, 2015. Available from: <http://www.ameli.fr/assures/soins-et-remboursements/combien-serez-vous-rembourse/consultations/les-consultations-en-metropole/dans-le-cadre-du-parcours-de-soins-coordonnes.php>.

14. *Ontario drug benefit formulary/Comparative drug index*. Ministry of Health and Long Term Care, 2015. Available from: <https://www.healthinfo.moh.gov.on.ca/formulary/>.

15. *Ontario drug benefit: Dispensing fees*. Ministry of Health and Long Term Care, 2015. Available from: <http://www.health.gov.on.ca/en/public/programs/drugs/programs/odb/opdp_dispensing_fees.aspx>.

16. *List of medications*. Régie de l'assurance maladie Québec, 2015. Available from: <http://www.ramq.gouv.qc.ca/en/regie/legal-publications/Pages/list-medications.aspx>.

17. *British national formulary*. MedicinesComplete, 2015. Available from: <https://www.medicinescomplete.com/mc/bnf/current/>.

18. *Lauer-Fischer*. 2017 [cited 2017 June 6]. <https://www.lauer-fischer.de/LF/Seiten/Verwaltung/Kundencenter/1.aspx2017>

19. Fischer, L. *Lauer-Taxe*. Lauer Fischer, 2015. Available from: <https://www.lauer-fischer.de/LF/Seiten/Verwaltung/Kundencenter.aspx>.

20. Health, M.o.S.A.a. *Prix et tarif des medicaments (specialites pharmaceutiques)*. Ministry of Social Affairs and Health, 2015. Available from: <http://www.sante.gouv.fr/prix-et-tarif-des-medicaments-specialites-pharmaceutiques.html>.

21. *Fees for dispensation*. Ministry of Social Affairs and Health, 2015. Available from: <http://www.sante.gouv.fr/mise-en-place-d-honoraires-de-dispensation-en-officine.html>.

22. *Schedule of benefits for laboratory services*. Ministry of Health and Long-Term Care, 2013. Available from: <http://www.health.gov.on.ca/english/providers/program/ohip/sob/lab/lab_services_sched_01_19990401.pdf>.

23. *Tarifs de la nomenclature des actes et services des laboratoires (lab costs)*. Securite Sociale Assurance Maladie, 2012. Available from: <http://www.cns.lu/files/listepos/Tarifs_Lab20120101.pdf>.
