# Supplementary material for: The cost of Mycobacterium avium complex lung disease in Canada, France, Germany, and the United Kingdom: a nationally representative observational study
Source: BMC Health Serv Res. 2018 Sep 10;18:700. doi: 10.1186/s12913-018-3489-8 (PMC6131733; doi:10.1186/s12913-018-3489-8)
Supplement: Supplementary file 3 — Exploratory analysis 1. Supplemental methods and results of the first exploratory analysis, comparing direct medical costs while having positive sputum cultures, versus costs from the time of first negative sputum culture. (DOCX 18 kb) [file 12913_2018_3489_MOESM3_ESM.docx]

**Additional file 3: Exploratory Analysis 1**

The 2 year resource collection period was split for each patient into time while having positive sputum cultures, and time from the first negative sputum culture onward. This second phase began on the date that patients had a negative test result that a physician deemed a true negative culture conversion with no positive test results following. If there are positive tests after a negative test result the next negative test was checked.

Resource use items were then categorized by occurring before or after a negative culture conversion; before the culture conversion patients’ resource use was classified as occurring while positive, and after the culture conversion the resource use was classified as occurring while testing negative. Costs in each category were averaged to an average cost per month. As in the regression analysis, costs were compared using the UK unit costs, allowing for comparisons to include the entire population that was included.

The exploratory analysis was performed only on patients that had time classified as both NTMLD positive and negative during the 2 year resource collection window; a sample of 44 patients from the larger NTMLD group met this criteria.

The average cost per month (30 days) for patients when positive was more than double the cost when the same patients were negative. Hospitalizations, physician visits, pulmonary exacerbations, and lab tests drove this difference, though every category of cost was higher in the positive state. Treatment costs were higher in the positive health state, but not by a large amount. Median monthly costs were 3 times higher per month in the positive health state than the negative health state.

Table 1. Monthly resource use costs for patients while testing positive versus while testing negative for NTMLD

| **Health care resource** | **While testing positive for NTMLD** | | **While testing negative for NTMLD** | |
| --- | --- | --- | --- | --- |
|  | **Average cost per month (2015 £)** | **SD** | **Average cost per month (2015 £)** | **SD** |
| Hospital use |  |  |  |  |
| ER visits | 3 | 13 | 1 | 3 |
| Hospitalizations | 126 | 201 | 27 | 88 |
| Health care professionals |  |  |  |  |
| Physicians | 53 | 87 | 26 | 30 |
| Infusion clinics | 2 | 14 | 1 | 9 |
| Pulmonary exacerbation | 51 | 123 | 24 | 66 |
| Laboratory and diagnostic tests | 62 | 55 | 32 | 49 |
| Medications* | 60 | 107 | 44 | 78 |
| Total costs (mean, SD) | 358 | 328 | 153 | 171 |
| Total costs (median, range) | 286 | 43 - 1423 | 94 | 0 - 717 |

*Abbreviations: SD = standard deviation; NTMLD = nontuberculous mycobacterial lung disease*
