# Supplementary material for: The cost of Mycobacterium avium complex lung disease in Canada, France, Germany, and the United Kingdom: a nationally representative observational study
Source: BMC Health Serv Res. 2018 Sep 10;18:700. doi: 10.1186/s12913-018-3489-8 (PMC6131733; doi:10.1186/s12913-018-3489-8)
Supplement: Supplementary file 4 — Exploratory analysis 2. Supplemental methods and results of the second exploratory analysis involving cost regression models. (DOCX 25 kb) [file 12913_2018_3489_MOESM4_ESM.docx]

**Additional file 4: Exploratory Analysis 2**

The data used in the linear regressions was un-weighted and costs for all countries were calculated using UK unit costs so that differences in unit costing by country would not be the main source of cost variability.

In the simple linear regression, variables thought to be potentially associated with the total cost of NTMLD management were regressed against the total cost (UK £) transformed on the log10 scale. Significant predictors of cost included: presence of bronchiectasis vs. no bronchiectasis); an unknown result in a chest radiograph (vs. negative result); an unknown result in an HRCT scan (vs. negative result); a physician specializing in infectious disease (vs. general medicine); a physician specializing in pulmonology or respiratory medicine (vs. general medicine); patients in France (vs. patients treated in Canada; borderline significant, *p*=0.055); weight, and; date of symptom onset. (Table 1) The latter variable, date of symptom onset, captured the prevalence-based feature of the study design, such that included patients could be incident prior to or during the resource utilization observation window. This variable was statistically significant and was used as an adjustment factor in the multiple linear regression.

In the multiple linear regression, characteristics found significant in linear regression were combined with date of symptom onset, which was highly significant in the simple linear regression and potentially confounding the relationship between other variables and overall cost. In the multiple linear regression, we found that only physician specialty remained significant, with both physicians specializing in infectious disease and in pulmonology or respiratory medicine decreasing total cost compared to those in general medicine. (Table 2).

For all tables, the interpretation of the coefficient for categorical variables is the increase in log10 cost for the category listed relative to the reference category (listed as “vs. xxx”). Positive coefficients represent increased costs whereas negative coefficients represent decreased costs. Coefficients for continuous variables are interpreted as the increase in log10 cost per unit increase of that variable. A p-value less than 0.05 is highlighted in red, indicating that the variable is statistically significant in its association with cost (log10 transformed).

Rows are shaded to show groupings of multiple levels from a single categorical variable. E.g. in Table 1, there were three levels for smoking status: smoker, non-smoker, and unknown. Smoker was selected as the reference treatment, and the relative cost among non-smokers vs. smokers and among those of unknown smoking status vs. smokers are presented in the seventh and eighth rows, both shaded in grey to indicate they belong to the same category.

Table 1: Simple linear regression of select characteristics against total annual NTMLD related cost (all expressed in £), transformed on log10 scale.

| **Covariate** | **Coefficient** | **SE** | **P-value** |
| --- | --- | --- | --- |
| *M. avium* (vs. absence of *M. avium*) | 0.133 | 0.101 | 0.187 |
| *M. intracellulare* (vs. absence of *M. intracellulare*) | -0.176 | 0.100 | 0.079 |
| *M. chimaera* (vs. absence of *M. chimaera*) | -0.059 | 0.280 | 0.834 |
| Other NTM species (vs. absence of other NTM species) | 0.016 | 0.438 | 0.970 |
| Remains positive (vs. converted to negative) | 0.098 | 0.098 | 0.320 |
| Female sex (vs. male) | -0.042 | 0.109 | 0.700 |
| Non-smoker (vs. smoker) | -0.022 | 0.115 | 0.849 |
| Unknown smoking status (vs. smoker) | -0.452 | 0.256 | 0.079 |
| Bronchiectasis (vs. absence of bronchiectasis) | -0.303 | 0.098 | 0.002 |
| No private insurance (vs. privately insured) | 0.013 | 0.127 | 0.921 |
| Unknown private insurance (vs. privately insured) | -0.124 | 0.154 | 0.420 |
| Not referred for social services (vs. referred) | 0.013 | 0.127 | 0.921 |
| Unknown if referred for social services (vs. referred) | -0.124 | 0.154 | 0.420 |
| Nodular or cavitary opacities on chest radiograph (vs. none) | 0.078 | 0.139 | 0.576 |
| Unknown if nodular or cavitary opacities on chest radiograph (vs. none) | -0.284 | 0.140 | 0.044 |
| HRCT scan shows multifocal bronchiectasis with multiple small nodules (vs. none) | 0.009 | 0.122 | 0.944 |
| Unknown if HRCT scan shows multifocal bronchiectasis with multiple small nodules (vs. none) | -0.355 | 0.119 | 0.003 |
| HRCT scan shows cavities/nodular change (vs. none) | -0.118 | 0.121 | 0.332 |
| Unknown if HRCT scan shows cavities/nodular change (vs. none) | -0.411 | 0.110 | <0.001 |
| Infectious disease (vs. general medicine) | -0.433 | 0.168 | 0.011 |
| Internal medicine (vs. general medicine) | -0.292 | 0.156 | 0.062 |
| Pulmonology/respiratory medicine (vs. general medicine) | -0.374 | 0.148 | 0.012 |
| Other (vs. general medicine) | 0.271 | 0.444 | 0.542 |
| Single specialty group private (vs. solo private practice) | 0.090 | 0.464 | 0.846 |
| Multi specialty group private (vs. solo private practice) | 0.144 | 0.474 | 0.761 |
| Hospital-based outpatient clinic (vs. solo private practice) | -0.043 | 0.453 | 0.924 |
| University-based research/teaching hospital (vs. solo private practice) | 0.186 | 0.441 | 0.674 |
| Community-based research/teaching hospital (vs. solo private practice) | 0.206 | 0.455 | 0.651 |
| Germany (vs. Canada) | 0.088 | 0.144 | 0.540 |
| France (vs. Canada) | 0.309 | 0.160 | 0.055 |
| UK (vs. Canada) | 0.118 | 0.124 | 0.343 |
| Age at diagnosis (per year increase) | 0.000 | 0.003 | 0.915 |
| Weight (per kg increase) | 0.010 | 0.004 | 0.004 |
| Date of symptom onset (per day increase) | 0.001 | 0.000 | <0.001 |

*Abbreviations: HRCT = high resolution computed tomography; NTM = non-tuberculosis mycobacterium; SE = standard error; UK = United Kingdom; vs. = versus*

Table 2: Multiple linear regression of select characteristics combined with date of symptom onset, against total annual NTMLD related cost (expressed in £) transformed on log10 scale.

| **Covariate** | **Coefficient** | **SE** | **P-value** |
| --- | --- | --- | --- |
| Date of symptom onset (per day increase) | 0.001 | 0.000 | <0.001 |
| Bronchiectasis (vs. no bronchiectasis) | -0.150 | 0.086 | 0.081 |
| Date of symptom onset (per day increase) | 0.001 | 0.000 | <0.001 |
| Nodular or cavitary opacities on chest radiograph (vs. none) | 0.021 | 0.120 | 0.863 |
| Unknown if nodular or cavitary opacities on chest radiograph (vs. none) | 0.037 | 0.129 | 0.775 |
| Date of symptom onset (per day increase) | 0.001 | 0.000 | <0.001 |
| HRCT scan shows multifocal bronchiectasis with multiple small nodules (vs. none) | 0.022 | 0.106 | 0.839 |
| Unknown if HRCT scan shows multifocal bronchiectasis with multiple small nodules (vs. none) | 0.052 | 0.118 | 0.664 |
| Date of symptom onset (per day increase) | 0.001 | 0.000 | <0.001 |
| HRCT scan shows cavities/nodular change (vs. none) | -0.079 | 0.106 | 0.454 |
| Unknown if HRCT scan shows cavities/nodular change (vs. none) | 0.002 | 0.112 | 0.985 |
| Date of symptom onset (per day increase) | 0.001 | 0.000 | <0.001 |
| Infectious disease (vs. general medicine) | -0.377 | 0.140 | 0.008 |
| Internal medicine (vs. general medicine) | -0.184 | 0.130 | 0.161 |
| Pulmonology/respiratory medicine (vs. general medicine) | -0.299 | 0.123 | 0.017 |
| Other (vs. general medicine) | 0.098 | 0.370 | 0.791 |
| Date of symptom onset (per day increase) | 0.001 | 0.000 | <0.001 |
| Germany (vs. Canada) | 0.160 | 0.120 | 0.186 |
| France (vs. Canada) | 0.201 | 0.134 | 0.138 |
| UK (vs. Canada) | 0.094 | 0.104 | 0.366 |
| Date of symptom onset (per day increase) | 0.000 | 0.000 | <0.001 |
| Weight (per kg increase) | 0.004 | 0.003 | 0.266 |

*Abbreviations: HRCT = high resolution computed tomography; SE = standard error; UK = United Kingdom; vs. = versus*
